# Resveratrol reduces muscle atrophy and stress pathway activation in a combined disuse-hypoxia-mouse model

**DOI:** 10.3389/fphar.2026.1753486

**Published:** 2026-02-12

**Authors:** Aziz Ahmad Khan, Sajid Khan Sadozai, Fawad Ali, Nemat Khan, Rizwan Qaisar

**Affiliations:** 1 Department of Pharmacy, Kohat University of Science and Technology, Kohat, Pakistan; 2 Department of Medical Sciences, Khalifa University, Abu Dhabi, United Arab Emirates; 3 Centre for Biotechnology, Khalifa University, Abu Dhabi, United Arab Emirates; 4 School of Biomedical Sciences, Faculty of Health, Medicine and Behavioural Sciences, The University of Queensland, Brisbane, QLD, Australia; 5 Department of Basic Medical Sciences, College of Medicine, University of Sharjah, Sharjah, United Arab Emirates; 6 Research Institute of Medical and Health Sciences, University of Sharjah, Sharjah, United Arab Emirates

**Keywords:** apoptosis, endoplasmic reticulum (ER) stress, hindlimb unloading, muscle atrophy, muscle strength, necroptosis, skeletal muscle

## Abstract

**Introduction:**

Mechanical disuse and hypoxia synergistically worsen muscle atrophy by activating apoptosis, necroptosis, and ER stress pathways. While resveratrol, a natural polyphenol, has shown protective effects in isolated disuse or hypoxia models, its efficacy under combined conditions remains unclear.

**Methods:**

Male C57BL/6J mice (4 months old) were assigned to ground control or hindlimb unloading (HU) groups under normoxia (21% O_2_) or hypoxia (15% O_2_) and treated daily with placebo or resveratrol (20 or 40 mg/kg) for two weeks. Muscle mass, grip strength, cling time, and gene expression of apoptotic, necroptotic, and ER stress markers were assessed.

**Results:**

HU-hypoxia significantly reduced muscle mass and function, with upregulation of stress-related genes. Resveratrol showed dose-dependent protection: 20 mg/kg modestly reduced atrophy, while 40 mg/kg nearly preserved muscle mass and strength to control levels under both normoxic and hypoxic conditions. To our knowledge, this is the first study to demonstrate protective effects of resveratrol in a combined HU and hypoxia model of muscle atrophy, accompanied by modulation of apoptosis, necroptosis and ER stress related gene expression.

**Conclusion:**

These results suggest that resveratrol may decrease muscle degradation in fast twitch dominant muscles under combined disuse and hypoxia. However, these results are restricted to gastrocnemius muscle in mice, and further investigations in slow twitch muscles and clinical models are required before clinical relevance can be confirmed. These findings support its potential as a therapeutic agent for muscle loss in clinical and spaceflight settings, warranting further translational research.

## Graphical Abstract

**Figure:**
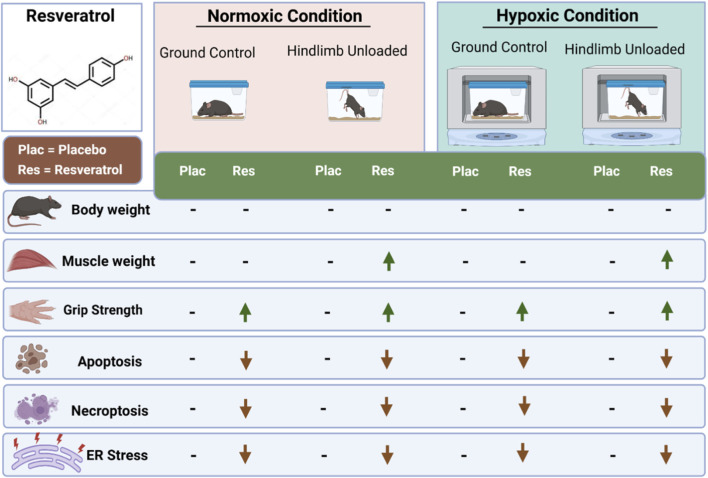

## 1 Introduction

Long periods of immobility are too much for the human body to adjust to. Medical conditions like stroke, bone fracture, or major organ failure can make it hard to move around and make you stay in bed, which can lead to several physiological problems (Cruz-Jentoft and Sayer, 2019). Muscle atrophy is one of the consequences of prolonged immobility. The lack of mechanical use leads to rapid loss in both muscle mass and strength.

Hypoxia (low oxygen levels), in conditions such as congestive heart failure (CHF) and chronic obstructive pulmonary disease (COPD), further aggravates muscle atrophy by triggering protein breakdown pathways and activating cellular stress responses, including endoplasmic reticulum stress (ER stress) (Sakuma and Yamaguchi, 2012; Puthucheary et al., 2013; Debevec et al., 2018; Nguyen, Choi, and June 2020). Altered expression of anti-apoptotic regulators such as B-cell lymphoma 2 (BCL2), Increased levels of pro-apoptotic genes such as Bcl-2-associated X protein (BAX) and activation of inflammatory cell death mediators including caspase-1, as well as necroptosis related kinases receptor interacting protein kinase 1 and 3 (RIPK1/3) and mixed lineage kinase domain-Like pseudo kinase (MLKL) have been reported in muscle under disuse or hypoxic conditions. ER stress markers such as activating transcription factor 4 (ATF4) and X-box binding protein 1 (XBP1) are also upregulated in these settings. Together, these stress pathways contribute to muscle wasting during conditions of reduced mechanical loading and low oxygen availability (Khan et al., 2025; Jeong et al., 2025).

At present, there are no approved pharmacological interventions that prevent disuse related muscle atrophy in clinical settings. Moreover, experimental interventions involving human subjects are constrained by ethical and practical considerations, thereby necessitating the use of animal models.

The hindlimb unloaded (HU) mouse model is one of the most common experimental models used to mimic the effects of long-term immobility on the body (Momken et al., 2011) In this model, mice are prevented from bearing weight on their hindlimbs by being suspended by their tails. As a result, the hindlimbs are mechanically unloaded, and bodily fluids are directed towards the brain, impairing the structural and functional integrity of muscles and other organs. We have extensively utilized this well-established model to mimic the conditions of disuse in our previous studies (Khan et al., 2022; Al-Daghestani et al., 2024; Ranade et al., 2023; Siddiqui et al., 2022). Moreover, we recently adapted this model to incorporate systemic hypoxia, making it more suitable for simulating immobilization under reduced oxygen availability (Khan et al., 2025).

Resveratrol, a natural polyphenol found in peanuts, cherries, and grapes, possesses various biological properties (Chunxiao et al., 2025), including antioxidative, anti-apoptotic, and muscle protein regenerative effects (Bastin and Djouadi, 2016). Previous studies have shown that resveratrol can attenuate muscle wasting in various atrophic conditions, including microgravity analogs and unloading models, and can improve recovery of muscle function (Mortreux et al., 2019; Bennett et al., 2013). Moreover, it has been found to reduce ER stress and improving muscle function in HU mice (Toniolo et al., 2023). However, its effects have not been evaluated in a combined model where disuse and hypoxia occur simultaneously, conditions that may more closely represent prolonged hospitalization or reduced oxygen exposure.

This knowledge gap is addressed in this study, where we investigated the effects of resveratrol in a combined hindlimb unloading and hypoxia model in a dose dependent manner, with a focus on apoptosis, necroptosis, and ER stress related gene expression. We predicted that the negative effects of HU and hypoxia in these mice would be partially mitigated by resveratrol.

## 2 Materials and methods

### 2.1 Experimental protocol

**FIGURE 1:**
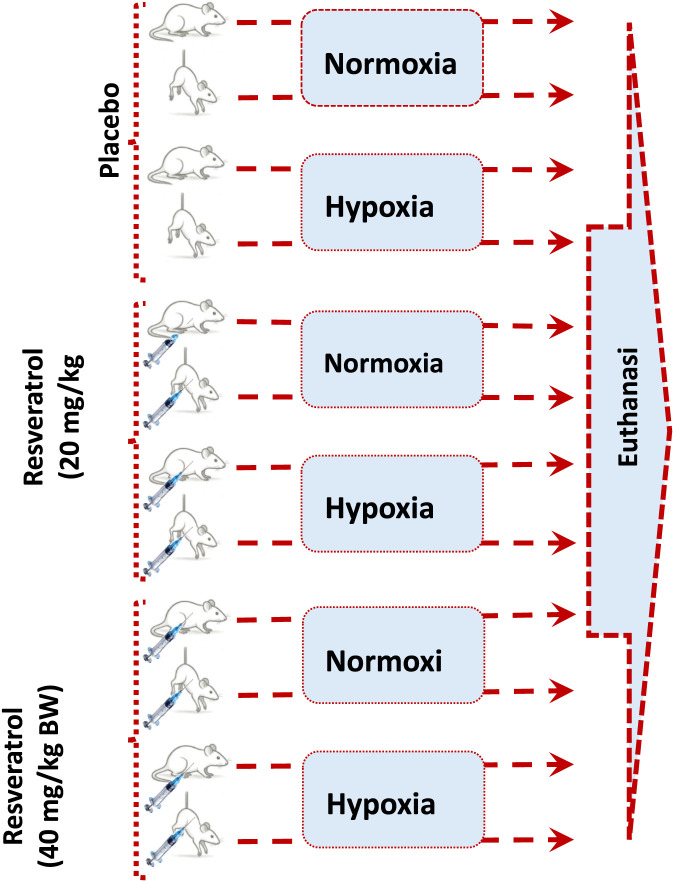
The study design.

Total 36 male, 4-month-old, C57BL/6J mice were randomly divided into normoxic and hypoxic groups, and further subdivided them into ground-controls and HU mice, which were further subdivided into three groups based on intraperitoneal administration of placebo vehicle, resveratrol 20 mg/kg, and resveratrol 40 mg/kg, result in total of twelve subgroups (Figure 1), with 3 mice in each subgroup. Only male mice were included to reduce variability associated with estrous phase dependent physiological changes. The doses of resveratrol were selected based on previous studies (Akosman et al., 2019; Wang et al., 2022).

The hypoxic condition was produced by placing the cages in a hypoxia chamber with 15% oxygen levels (FIC-30-1/HYPO, PLAS LABS, Michigan, United States), and the normoxic group was kept in a chamber with normal oxygen concentration, i.e., 21%, for the complete experimental duration of 14 days. The choice of 15% oxygen is based on established models of systemic hypoxia relevant to cardiopulmonary disease and rodent hypoxia research.

The HU model was achieved by suspending the mice from the tail at an angle of 30° from the floor, ensuring that the forelimbs remain touching the floor of the cage while the hindlimbs remain elevated from the floor for the complete duration of the experiment, with daily monitoring for stress and movement (Zhang et al., 2018). Resveratrol was prepared in 10% dimethyl sulfoxide (DMSO) in sterile 0.9% saline, filtered through a 0.22 μm membrane, and administered intraperitoneally once daily at 20 or 40 mg/kg for the entire experimental period. Control groups received an equal volume of vehicle (10% DMSO in saline) (Alaasam et al., 2024).

A controlled environmental condition (with light/dark periods of 12 h each, 22 °C ± 1 °C) was provided to all groups, with food (standard chow diet for mice) and water provided *ad-libitum*. After the completion of the experiment, the gastrocnemius muscles were excised, weighed, and processed for subsequent analysis. The experimental procedures were approved by the University of Sharjah Animal Care and Use Committee (ACUC-08-09-2023), in agreement with accepted international guidelines and regulations.

### 2.2 Grip strength measurement

Digital grip strength meter (Columbus Instruments, Columbus, OH) was used to measure grip strength. Mice were allowed to grasp the grid of the grip meter with their limbs and were gently pulled back horizontally until release. The maximum force shown in the meter was recorded. The exact process was repeated ten times for each animal, with at least 1 min of rest between trials. The grip strength for two fore limbs and all four limbs was measured the same way, and the mean of the peak three readings was designated as the final grip strength of mice. To compare the inter-individual grip strength, these recordings were normalized to body weight according to specified guidelines (Khan et al., 2022; Ibrahim et al., 2022).

### 2.3 Cling time test

We measured the muscle strength by a modified wire-hang (cling time) test, described in the JoVE method (Deacon, 2013). Mice were held from the middle or base of the tail and inverted with the stomach down to grasp the wire mesh with a weight placed on the table. As it holds the wire mesh, raises the mice, and starts the stopwatch until it releases the weight in 3 s, the time it holds the weight is noted. Each subject repeated the exact three times after an interval of 5-10 s. And the mean of the maximum two recordings was normalized with body weight for further analysis (Qaisar et al., 2025).

### 2.4 RNA extraction and quantitative real-time PCR validation

Total RNA was extracted from snap-frozen gastrocnemius muscle with TRIzol reagent (Invitrogen). RNA integrity was evaluated by the agarose gel method, while RNA concentration was measured with a NanoDrop spectrophotometer (Thermo Scientific, United States). cDNA was synthesized by reverse transcription (RT) (Promega, Madison, WI, United States), while the 7900HT fast real-time polymerase chain reaction (PCR) System (Applied Biosystems, Inc., Foster City, CA, United States was used for the assay of quantitative real-time PCR. The detection and quantification of the expression levels of targeted genes were performed by Real-time Master Mix (Toyobo, Osaka, Japan). The PCR reaction consists of an initial denaturation at 95 °C for 10 min followed by 40 cycles at 95 °C for 30 s, 60 °C for 10 s, and 72 °C for 30 s, and finally 72 °C for 10 min. Amplification of the target cDNA was normalized to β-actin expression (Yuan et al., 2015). The corresponding primers used in this study are listed in Supplementary Table S1. Relative expression levels were calculated using 2^(-ΔΔCt)^ method (Alamdari et al., 2012; Zhang et al., 2019).

### 2.5 Statistical analysis

All numerical data are presented as mean ± standard deviation (SD), and comparisons among groups of mice were performed using one-way analysis of variance (ANOVA) followed by Tukey’s multiple-comparison test. Gene expression data were analysed using one-way ANOVA with Tukey’s *post hoc* comparisons to identify differences among various groups. Statistical analysis was performed using GraphPad Prism 10 (GraphPad Software, La Jolla, CA), and p < 0.05 was considered statistically significant.

## 3 Results

### 3.1 Body and muscle mass

**FIGURE 2:**
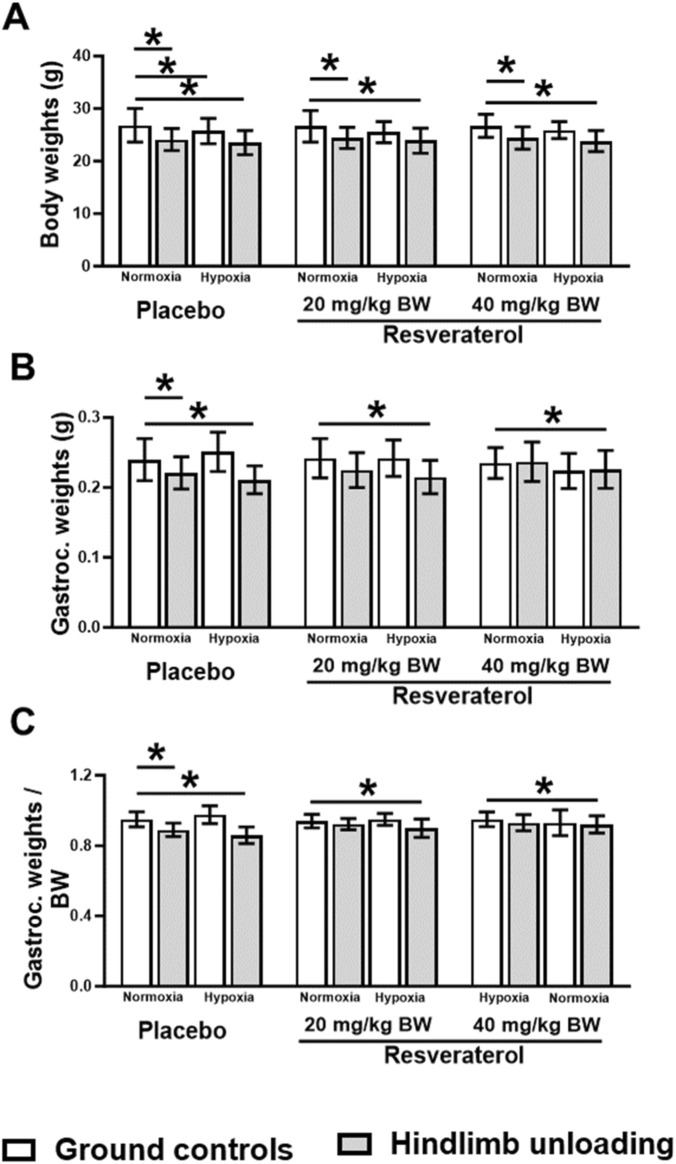
Body weights **(A)**, and absolute **(B)** and relative **(C)** gastrocnemius muscle weights in GC and HU mice under normoxia and hypoxia among placebo- and resveratrol-treated groups. One-way analysis of variance, *p < 0.05 compares the placebo mice with resveratrol-treated groups, at similar loading and oxygen condition. (GC; Ground-based Controls, HU; Hindlimb Unloaded mice).

We investigated absolute and relative muscle and body weight in resveratrol-treated and placebo groups under hypoxic and normoxic conditions. No significant differences in body weight were observed between resveratrol-treated and placebo groups under normoxic conditions. A slight reduction in body weight was noted across all experimental groups; however, this decrease was not statistically significant (Figure 2A).

The loss of gastrocnemius muscles mass, a typical feature observed in HU, was treated with two different doses of resveratrol. A dose-dependent effect on preservation was observed. The 20 mg dose did not significantly reverse the muscle loss. However, increasing the dose to 40 mg/kg resulted in a substantial preservation of muscle mass, particularly in the HU groups. In these groups, muscle mass increased to 0.237 g in HU-normoxia and 0.226 g in HU-hypoxia, compared to 0.221 g and 0.211 g in the placebo-treated groups, respectively. These findings suggest that 40 mg/kg dose effectively preserved muscle mass in both normoxic and hypoxic HU mice, with muscle weights approaching those observed in GC control groups (Figure 2B).

When muscle weight was normalized to body weight, the results reflected a similar trend: the placebo-treated HU group showed significant muscle loss without (p = 0.014) or with (p = 0.01) hypoxia, while the resveratrol-treated groups, especially those treated with 40 mg/kg dose, demonstrated a notable prevention of muscle atrophy. This protective effect was most evident in HU-hypoxic and HU-normoxic mice, indicating that resveratrol’s ability to counter muscle loss was more effective under these conditions (Figure 2C).

### 3.2 Muscle strength and endurance

**FIGURE 3:**
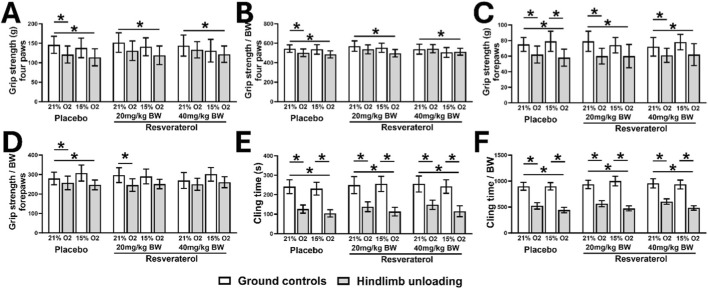
Absolute **(A)** and relative **(B)** grip strengths of four paws, absolute **(C)** and relative **(D)** grip strengths of forepaws, and absolute **(E)** and relative **(F)** cling times in GC and HU mice under 21% and 15% oxygen among placebo- and resveratrol-treated groups. One-way analysis of variance, *p < 0.05 placebo mice with similar loading and oxygen status. (GC; Ground-based Controls, HU; Hindlimb Unloaded mice).

The function of the skeletal muscle was evaluated using Digital Grip Strength meter. We observed significantly lower grip strengths of four paws (related to all four limbs) in GC-hypoxia than GC-normoxia mice in placebo-treated groups (p < 0.001). Conversely, treatment with resveratrol prevented this reduction, irrespective of the dosing amount (Figure 3A). However, such effects of resveratrol were not observed in HU mice with or without hypoxia. When normalized to body weights, we observed a significant reduction in grip strength in GC-hypoxia than in GC-hypoxia mice (p < 0.05), which was not observed in the HU mice (Figure 3B).

We also observed lower grip strengths of forepaws (related to the frontal two limbs) in GC-hypoxia mice compared to GC-normoxia mice in the mice treated with placebo (p < 0.05), or 20 mg (p < 0.05) and 40 mg (p < 0.05) of resveratrol. Similarly, HU-hypoxia mice also exhibited lower forepaw grip strength than HU-normoxia mice in placebo-treated group (p < 0.05) (Figure 3C). However, treatment with resveratrol prevented this reduction of grip strength in HU-hypoxia mice, when compared to HU-normoxia mice. When normalized to body weights, the grip strengths of forepaws was significantly lower in HU-hypoxia than in GC-normoxia mice (p = 0.012) (Figure 3D). However, this difference was not observed in the resveratrol-treated groups.

We also observed a significant reduction in the absolute cling time in HU-hypoxia than in HU-normoxia mice in the mice treated with placebo (p < 0.001), 20 mg (p < 0.001), or 40 mg (p < 0.001) of resveratrol (Figure 3E). Similar trends were observed in the relative cling time normalized to body weights (Figure 3F).

### 3.3 Expressions of apoptosis, necroptosis and ER stress markers

**FIGURE 4:**
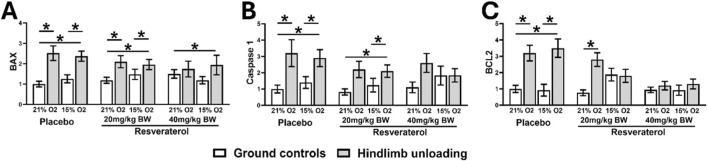
Relative mRNA expressions of BAX **(A)**, Caspase 1 **(B)**, and BCL2 **(C)** in GC and HU mice under 21% and 15% oxygen among placebo- and resveratrol-treated groups. One-way analysis of variance, *p < 0.05 compares the placebo mice with resveratrol-treated groups, at similar loading and oxygen condition. (GC; Ground-based Controls, HU; Hindlimb Unloaded mice, BAX; BCL2-associated X, apoptosis regulator, BCL2; B-cell lymphoma 2).

The dose-dependent effect of resveratrol on mRNA expression levels of apoptosis-related markers (BAX, caspase-1 and BCL2) was evaluated. qPCR analysis described that compared to HU-normoxic mice, the HU-hypoxic mice had higher expressions of BAX (p < 0.001), caspase-1 (p = 0.01), and BCL2 (p < 0.001) (Figures 4A–C). 20 mg of resveratrol treatment reduced the expression of BCL2 in HU-hypoxic mice similar to the HU-normoxic mice (Figure 4C). However, similar effects were not observed for the expressions of BAX (Figure 4A) and caspase 1 (Figure 4B). Conversely, treatment with 40 mg of resveratrol reduced the expressions of all three genes in the HU-hypoxic mice to the levels of HU-normoxic mice (Figures 4A–C).

**FIGURE 5:**
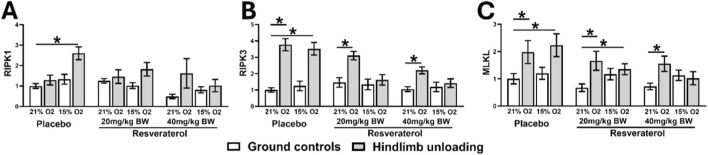
Relative mRNA expressions of RIPK1 **(A)**, RIPK3 **(B)**, and MLKL **(C)** in GC and HU mice under 21% and 15% oxygen among placebo- and resveratrol-treated groups. One-way analysis of variance, *p < 0.05, compares the placebo mice with resveratrol-treated groups, at similar loading and oxygen condition. (GC; Ground-based Controls, HU; Hindlimb Unloaded mice, RIPK1; Receptor-interacting serine/threonine-protein kinase 1, RIPK3; Receptor-interacting serine/threonine-protein kinase 3, MLKL; Mixed lineage kinase domain-like pseudo kinase).

Next, the expressions of necroptotic signaling genes, including RIPK1, RIPK3, and MLKL, were higher in HU-hypoxic than in GC-normoxic mice in the placebo-treated groups (Figures 5A–C). However, similar effects were not observed for RIPK1 expressions in both groups of resveratrol treatments. Resveratrol also reduced the expressions of RIPK3 (Figure 5A), RIPK3 (Figure 5B), and MLKL (Figure 5C) in HU-hypoxia mice, which were similar to GC-normoxia and HU-normoxia mice.

**FIGURE 6:**
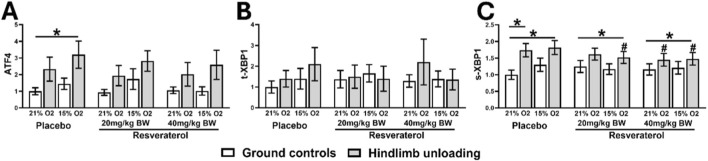
Relative mRNA expressions of ATF4 **(A)**, t-XBP1 **(B)**, and sXBP1 **(C)** in GC and HU mice under 21% and 15% oxygen among placebo- and resveratrol-treated groups. One-way analysis of variance, *p < 0.05, compares the placebo mice with resveratrol-treated groups, at similar loading and oxygen condition. (GC; Ground-based Controls, HU; Hindlimb Unloaded mice, ATF4; Activating Transcription Factor 4, t-XBP1; total x-box binding protein 1, s-XBP1; spliced x-box binding protein 1).

Lastly, the ER stress response markers, including ATF4, total XBP1 (tXBP1), and spliced XBP1 (s-XBP1), were significantly elevated in placebo-treated HU-hypoxia mice. Resveratrol at both doses significantly reduced the expressions of these genes, suggesting attenuation of ER stress responses in this model (Figures 6A–C).

## 4 Discussion

We investigated the effects of resveratrol in a combined hindlimb unloading and hypoxia model, which represents conditions that may occur in patients with COPD, heart failure, prolonged hospitalization, or reduced oxygen exposure in settings such as high altitude and spaceflight. Our findings reveal several molecular changes in the skeletal muscle of HU-hypoxic mice compared to ground control. These changes highlight synergistic effects of disuse and hypoxia in the exacerbation of pathological processes in skeletal muscle, which is evident by the upregulation of genes responsible for apoptosis, necroptosis, and ER stress in these tissues of placebo-treated groups, which are consistent with previous studies on muscle atrophy (Gallot and Bohnert, 2021; Cheema et al., 2015). Although those studies examined age-related mitochondrial dysfunction or ER stress in different contexts, both studies reported activation of apoptosis and ER stress pathways similar to those observed in our combined disuse-hypoxia model. This convergence suggests that these molecular mechanisms are fundamental drivers of muscle atrophy across diverse conditions. The results of this study provide a prospective therapeutic option for the prevention and treatment of muscle atrophy caused by hypoxia and disuse by highlighting various biochemical pathways that can be inhibited by resveratrol in reducing muscle degeneration.

The use of a combined hypoxia and HU model is an essential step in the study of muscle atrophy. Prior research has primarily examined the impact of both factors separately (Yoshihara et al., 2021; Agrawal et al., 2018). But these dual-stressor models mimic clinical conditions in a better way, like extended immobilization experienced by hospitalized patients or the microgravity situations seen during space travel. This combined-model method also addresses a significant gap in current literature, which has primarily focused on single-induced models of atrophy, limiting our understanding of how various stressors interact to exacerbate muscle degeneration.

Various molecular mechanisms are involved in disuse- and hypoxia-induced muscle atrophy. The main causes of muscle degradation in atrophic disorders are increased apoptosis, necroptosis, and ER stress (Sartori, Romanello, and Sandri, 2021). The markers of Pro-apoptosis (BAX and caspase-1), necroptosis (RIPK1/3 and MLKL), and ER-stress-related genes (ATF4, XBP1) were all upregulated in HU-hypoxic in the placebo-treated group in this study, confirming their role in muscle degradation, which is consistent with previous research showing the critical role of these pathways in muscle loss (Yang et al., 2017; Afroze and Kumar 2019; Cheema et al., 2015).

However, resveratrol has significantly reduced the activation of these pathways, particularly at a dose of 40 mg/kg which suggests that it may be used as a multi-target treatment for muscle atrophy. With a marked reduction in the levels of BAX and caspase-1, two important apoptotic mediators, our data validates its well-established anti-apoptotic properties (Chen et al., 2013; Haramizu et al., 2017; Alam et al., 2023; Changizi et al., 2021). Additionally, it significantly inhibits necroptotic signaling, as demonstrated by the reduction in RIPK1, RIPK3, and MLKL levels. These findings are compelling, as necroptosis has been identified as a major regulator of cell death in skeletal muscle (Kamiya et al., 2022). Furthermore, resveratrol also downregulates ER stress pathways (Luo et al., 2023), as evident by a reduction in the level of ATF4 and XBP1 and BCL2. By targeting all these pathways, resveratrol may offer strong therapeutic effects compared to one that only focuses on a single mechanism of apoptosis or necrosis.

Our findings highlight a dose dependent response of resveratrol, with 40 mg per kg providing greater protection against muscle loss and functional decline than 20 mg per kg, emphasizing the importance of dose optimization. However, these findings are based on short term treatment in mice and may not fully represent the complexity of chronic human muscle atrophy. Protein level validation, histology, upstream pathway analysis (such as SIRT1, AMPK, and FoxO1), and long-term pharmacological studies are needed to further define mechanisms.

This study has various strengths including the dual stressor model that better reflects clinical and spaceflight conditions and the assessment of multiple molecular pathways relevant to muscle degeneration (Puthucheary et al., 2013; Debevec et al., 2018). However, this study has certain limitations, as intraperitoneal resveratrol administration may not accurately reflect its bioavailability or the optimal route of administration in humans. Additionally, the study was performed on male mice only, to reduce variability associated with estrous phase-dependent physiological changes. This limits the generalization of the findings to females, as sex-based differences in drug metabolism and response could exist. Moreover, conclusions are based on mRNA expression without confirmation at the protein or structural levels, and only male mice were included. Our analysis was restricted to the gastrocnemius muscle because its larger tissue mass enabled combined functional and molecular assessments from the same sample, which was essential given the limited number of animals. However, the soleus muscle, which is slow-twitch and highly oxidative, is classically more susceptible to disuse and hypoxia. Its omission limits the generalizability of our findings to fast-twitch dominant muscles. Future studies should include soleus to determine whether resveratrol provides similar protection in oxidative muscles, which are disproportionately affected in conditions of prolonged immobilization (Qaisar, Karim, and Elmoselhi, 2020). The focus of this study was on gross muscle size and accompanying molecular changes in cell stress pathways rather than histological architecture. Although fiber cross-sectional area and total protein content were not measured, previous work from our laboratory using similar demonstrates that reductions in muscle weight are consistently associated with decreased fiber cross-sectional area in HU alone (Khan et al., 2022; Ibrahim et al., 2022; Khan et al., 2025) or accompanied by hypoxia (Khan et al., 2025), confirming muscle fiber atrophy. These findings support the use of muscle weight as a valid indicator of atrophy in this experimental setting.

In summary, our study shows that resveratrol attenuates molecular and functional indicators of muscle atrophy in fast twitch dominant muscle induced by combined disuse and hypoxia. These findings extend previous work on resveratrol in single stressor models by showing protective effects in a dual stress setting that more closely reflects clinical and spaceflight conditions. However, these findings are limited to the gastrocnemius muscle, and further preclinical validation and confirmation for slow twitch phenotypes such as the soleus, as well as in clinical models is required before therapeutic translation. While further preclinical validation is required before therapeutic translation, our results position resveratrol as a biologically plausible and mechanistically relevant candidate for strategies targeting complex muscle wasting conditions.

## Data Availability

The raw data supporting the conclusions of this article will be made available by the authors, without undue reservation.

## References

[B1] Afroze D. Kumar A. (2019). ER stress in skeletal muscle remodeling and myopathies. FEBS Journal 286, 379–398. 10.1111/febs.14358 29239106 PMC6002870

[B2] Agrawal A. Rathor R. Kumar R. Suryakumar G. Ganju L. (2018). Role of altered proteostasis network in chronic hypobaric hypoxia induced skeletal muscle atrophy. PloS One 13, e0204283. 10.1371/journal.pone.0204283 30240405 PMC6150520

[B3] Akosman M. S. Türkmen R. Hüseyin Demirel H. Yeni D. Avdatek F. (2019). Protective effects of resveratrol on testicular oxidative stress induced of MK-801 in mice. Ank. Üniversitesi Veteriner Fakültesi Derg. 66, 171–176. 10.33988/auvfd.424703

[B4] Al-Daghestani H. Qaisar R. Al Kawas S. Ghani N. Rani K. G. A. Azeem M. (2024). pharmacological inhibition of endoplasmic reticulum stress mitigates osteoporosis in a mouse model of hindlimb suspension. Sci. Rep. 14, 4719. 10.1038/s41598-024-54944-7 38413677 PMC10899598

[B5] Alaasam E. R. Ali M. J. Al-Buthabhak K. M. Almudhafar R. H. Hadi N. R. Alexiou A. (2024). Nephroprotective role of resveratrol in renal ischemia-reperfusion injury: a preclinical study in sprague-dawley rats. BMC Pharmacol. Toxicol. 25, 82. 10.1186/s40360-024-00809-8 39468702 PMC11520524

[B6] Alam M. A. Caocci M. Ren Mi Chen Z. Liu F. Khatun M. S. (2023). Deficiency of caspase-1 attenuates HIV-1-associated atherogenesis in mice. Int. Journal Molecular Sciences 24, 12871. 10.3390/ijms241612871 37629052 PMC10454548

[B7] Alamdari N. Toraldo G. Aversa Z. Smith I. Castillero E. Renaud G. (2012). Loss of muscle strength during sepsis is in part regulated by glucocorticoids and is associated with reduced muscle fiber stiffness. Am. J. Physiol. Regul. Integr. Comp. Physiol. 303, R1090–R1099. 10.1152/ajpregu.00636.2011 23019215 PMC3517670

[B8] Bastin J. Djouadi F. (2016). Resveratrol and myopathy. Nutrients 8, 254. 10.3390/nu8050254 27136581 PMC4882667

[B9] Bennett B. T. Junaith S. M. Alway S. E. (2013). Effects of resveratrol on the recovery of muscle mass following disuse in the plantaris muscle of aged rats. PloS One 8, e83518. 10.1371/journal.pone.0083518 24349525 PMC3861503

[B10] Changizi Z. Moslehi A. Ali H. R. Eidi A. (2021). Chlorogenic acid induces 4T1 breast cancer tumor's apoptosis via p53, bax, Bcl‐2, and caspase‐3 signaling pathways in BALB/c mice. J. Biochem. Mol. Toxicol. 35, e22642. 10.1002/jbt.22642 33058431

[B11] Cheema N. Herbst A. McKenzie D. Aiken J. M. (2015). apoptosis and necrosis mediate skeletal muscle fiber loss in age‐induced mitochondrial enzymatic abnormalities. Aging Cell 14, 1085–1093. 10.1111/acel.12399 26365892 PMC4693455

[B12] Chen C. Jiang X. Zhao W. Zhang Z. Z. (2013). Dual role of resveratrol in modulation of genotoxicity induced by sodium arsenite via oxidative stress and apoptosis. Food Chem. Toxicol. 59, 8–17. 10.1016/j.fct.2013.05.030 23727334

[B13] Chunxiao L. Xin H. Bowen L. Bingqing S. Mengqi Z. Shuwen Z. (2025). Uncovering the mechanism of resveratrol in the treatment of asthma: a network pharmacology approach with molecular docking and experimental validation. Front. Pharmacol. 16, 1596737. 10.3389/fphar.2025.1596737 40453664 PMC12122464

[B14] Cruz-Jentoft A. J. Sayer A. A. (2019). Sarcopenia. Lancet 393, 2636–2646. 10.1016/S0140-6736(19)31138-9 31171417

[B15] Deacon R. M. J. (2013). Measuring the strength of mice. J. Visualized Experiments JoVE, 2610. 10.3791/2610 23770643 PMC3725666

[B16] Debevec T. Ganse B. Mittag U. Eiken O. Mekjavic I. B. Rittweger J. (2018). Hypoxia aggravates inactivity-related muscle wasting. Front. Physiology 9, 494. 10.3389/fphys.2018.00494 29867545 PMC5962751

[B17] Gallot Y. S. Bohnert K. R. (2021). Confounding roles of ER stress and the unfolded protein response in skeletal muscle atrophy. Int. Journal Molecular Sciences 22, 2567. 10.3390/ijms22052567 33806433 PMC7961896

[B18] Haramizu S. Asano S. Butler D. C. Stanton D. A. Hajira A. Mohamed J. S. (2017). Dietary resveratrol confers apoptotic resistance to oxidative stress in myoblasts. J. Nutritional Biochemistry 50, 103–115. 10.1016/j.jnutbio.2017.08.008 29053994 PMC5694367

[B19] Ibrahim Z. Ramachandran G. El-Huneidi W. Elmoselhi A. Qaisar R. (2022). Suppression of endoplasmic reticulum stress prevents disuse muscle atrophy in a mouse model of microgravity. Life Sci. Space Res. (Amst) 34, 45–52. 10.1016/j.lssr.2022.06.005 35940689

[B20] Jeong J. Y. Son Y. Kim Y. Lee N. Kim Y. Heo Y. J. (2025). Deferoxamine prevents dexamethasone-induced muscle atrophy by reducing MuRF1 and atrogin-1. Front. Pharmacol. 16, 1582216. 10.3389/fphar.2025.1582216 40703355 PMC12283682

[B21] Kamiya M. Mizoguchi F. Kawahata K. Wang D. Nishibori M. Day J. (2022). Targeting necroptosis in muscle fibers ameliorates inflammatory myopathies. Nat. Communications 13, 166. 10.1038/s41467-021-27875-4 35013338 PMC8748624

[B22] Khan A. A. Gul M. T. Karim A. Ranade A. Azeem M. Ibrahim Z. (2022). Mitigating sarcoplasmic reticulum stress limits disuse-induced muscle loss in hindlimb unloaded mice. NPJ Microgravity 8, 24. 10.1038/s41526-022-00211-w 35817772 PMC9273600

[B23] Khan S. A. Jose J. Ramachandran G. Rawas-Qalaji M. Ranade A. Karim A. (2025). Effects of concomitant hypoxia and simulated microgravity on skeletal muscle, liver, and lungs in a novel experimental mouse model. Acta Astronaut. 232, 580–587. 10.1016/j.actaastro.2025.04.010

[B24] Luo Y. Yang Z. Lai J. Wei L. Zhou G. Yu Y. (2023). [Retracted] resveratrol suppresses bupivacaine‐induced spinal neurotoxicity in rats by inhibiting endoplasmic reticulum stress via SIRT1 modulation. BioMed Res. Int. 2023, 1176232. 10.1155/2023/1176232 36865484 PMC9974252

[B25] Momken I. Stevens L. Bergouignan A. Desplanches D. Rudwill F. Chery I. (2011). Resveratrol prevents the wasting disorders of mechanical unloading by acting as a physical exercise mimetic in the rat. Faseb J. 25, 3646–3660. 10.1096/fj.10-177295 21715682

[B26] Mortreux M. Riveros D. Bouxsein M. L. Rutkove S. B. (2019). A moderate daily dose of resveratrol mitigates muscle deconditioning in a martian gravity analog. Front. Physiology 10, 899. 10.3389/fphys.2019.00899 31379604 PMC6656861

[B27] Nguyen T. T. N. Choi H. Jun H. S. (2020). Preventive effects of dulaglutide on disuse muscle atrophy through inhibition of inflammation and apoptosis by induction of Hsp72 expression. Front. Pharmacol. 11, 90. 10.3389/fphar.2020.00090 32153405 PMC7046759

[B28] Puthucheary Z. A. Rawal J. McPhail M. Connolly B. Ratnayake G. Chan P. (2013). Acute skeletal muscle wasting in critical illness. Jama 310, 1591–1600. 10.1001/jama.2013.278481 24108501

[B29] Qaisar R. Karim A. Elmoselhi A. B. (2020). Muscle unloading: a comparison between spaceflight and ground-based models. Acta Physiol. (Oxf) 228, e13431. 10.1111/apha.13431 31840423

[B30] Qaisar R. Srinivas M. Gul M. T. Ali Khan A. Ranade A. Jose J. (2025). Mesenchymal stem cell transplant as an intervention to ameliorate disuse-induced muscle atrophy in a mouse model of simulated microgravity. Acta Astronaut. 226, 275–282. 10.1016/j.actaastro.2024.10.060

[B31] Ranade A. Khan A. A. Gul M. T. Suresh S. Qaisar R. Ahmad F. (2023). Suppression of endoplasmic reticulum stress reverses hindlimb unloading-induced hepatic cellular processes in mice. Biochim. Biophys. Acta Gen. Subj. 1867, 130422. 10.1016/j.bbagen.2023.130422 37406741

[B32] Sakuma K. Yamaguchi A. (2012). Sarcopenia and cachexia: the adaptations of negative regulators of skeletal muscle mass. J. Cachexia, Sarcopenia Muscle 3, 77–94. 10.1007/s13539-011-0052-4 22476916 PMC3374017

[B33] Sartori R. Romanello V. Sandri M. (2021). Mechanisms of muscle atrophy and hypertrophy: implications in health and disease. Nat. Communications 12, 330. 10.1038/s41467-020-20123-1 33436614 PMC7803748

[B34] Siddiqui R. Qaisar R. Khan N. A. Alharbi A. M. Alfahemi H. Elmoselhi A. (2022). Effect of microgravity on the Gut microbiota bacterial composition in a hindlimb unloading model. Life (Basel) 12, 1865. 10.3390/life12111865 36431000 PMC9698145

[B35] Toniolo L. Concato M. Giacomello E. (2023). Resveratrol, a multitasking molecule that improves skeletal muscle health. Nutrients 15, 3413. 10.3390/nu15153413 37571349 PMC10421121

[B36] Wang R. Yuan W. Lu Li Lu F. Zhang L. Gong H. (2022). Resveratrol ameliorates muscle atrophy in chronic kidney disease via the axis of SIRT1/FoxO1. Phytotherapy Res. 36, 3265–3275. 10.1002/ptr.7499 35606908

[B37] Yang X.-Sa Yi T.-L. Zhang S. Xu Z.-W. Yu Z.-Qi Sun H.-T. (2017). Hypoxia-inducible factor-1 alpha is involved in RIP-induced necroptosis caused by *in vitro* and *in vivo* ischemic brain injury. Sci. Reports 7, 1–11. 10.1038/s41598-017-06088-0 28724891 PMC5517428

[B38] Yoshihara T. Natsume T. Tsuzuki T. Chang S.-W. Kakigi R. Machida S. (2021). Long-term physical inactivity exacerbates hindlimb unloading-induced muscle atrophy in young rat soleus muscle. J. Appl. Physiology 130, 1214–1225. 10.1152/japplphysiol.00494.2020 33600278

[B39] Yuan L. Han J. Meng Q. Xi Q. Zhuang Q. Jiang Yi (2015). Muscle-specific E3 ubiquitin ligases are involved in muscle atrophy of cancer cachexia: an *in vitro* and *in vivo* study. Oncol. Reports 33, 2261–2268. 10.3892/or.2015.3845 25760630

[B40] Zhang Ya N. Wen G. S. Jun Rui H. U. A. Feng X. Wen Jun W. E. I. Wang Ju F. (2018). Bone loss induced by simulated microgravity, ionizing radiation and/or ultradian rhythms in the hindlimbs of rats. Biomed. Environmental Sciences 31, 126–135. 10.3967/bes2018.015 29606191

[B41] Zhang S. Li R. Dong W. Yang H. Zhang Li Chen Y. (2019). RIPK3 mediates renal tubular epithelial cell apoptosis in endotoxin-induced acute kidney injury. Mol. Med. Rep. 20, 1613–1620. 10.3892/mmr.2019.10416 31257491 PMC6625383

