# Supplementary material for: Resveratrol reduces muscle atrophy and stress pathway activation in a combined disuse-hypoxia-mouse model
Source: Front Pharmacol. 2026 Feb 12;17:1753486. doi: 10.3389/fphar.2026.1753486 (PMC12936514; doi:10.3389/fphar.2026.1753486)
Supplement: Supplementary file 1 [file Table1.docx]

**Supplementary Table 1.** Primer sequences used in this study. (BAX; BCL2-associated X, apoptosis regulator, BCL2; B-cell lymphoma 2, RIPK1; Receptor-interacting serine/threonine-protein kinase 1, RIPK3; Receptor-interacting serine/threonine-protein kinase 3, MLKL; Mixed lineage kinase domain-like pseudo kinase, ATF4; Activating Transcription Factor 4, t-XBP1; total x-box binding protein 1, s-XBP1; spliced x-box binding protein 1).

| Gene | Forward | Reverse | References |
| --- | --- | --- | --- |
| BAX | AGACAGGGGCCTTTTTGCTA | AATT CGCCGGAGACACTCG | (Changizi et al. 2021) |
| BCL2 | CTTTGAGTTCGGTGGGGTCA | AGTTCCACAAAGGCATCCCA | (Changizi et al. 2021) |
| Caspase-1 | TTCAACATCTTTCTCCGAGGG | CACCTCTTTCACCATCTCCAG | (Alam et al. 2023) |
| RIPK1 | GACTGTGTACCCTTACCTCCGA | CACTGCGATCATTCTCGTCCTG | (Khan et al. 2025) |
| RIPK3 | TGGGCCTGCTAAGATGGCT | CTGCCAGAGTGTGGATTTGGT | (Zhang et al. 2019) |
| MLKL | CTGAGGGAACTGCTGGATAGAG | CGAGGAAACTGGAGCTGCTGAT | (Khan et al. 2025) |
| ATF4 | GGGTTCTGTCTTCCACTCCA | AAGCAGCAGAGTCAGGCTTTC | (Khan et al. 2025) |
| t-XBP1 | TGGCCGGGTCTGCTGAGTCCG | GTCCATGGGAAGATGTTCTGG | (Khan et al. 2025) |
| s-XBP1 | CTGAGTCCGAATCAGGTGCAG | GTCCATGGGAAGATGTTCTGG | (Khan et al. 2025) |
